# Supplementary material for: Identification of photoperiod-induced specific miRNAs in the adrenal glands of Sunite sheep (Ovis aries)
Source: Front Vet Sci. 2022 Jul 22;9:888207. doi: 10.3389/fvets.2022.888207 (PMC9354845; doi:10.3389/fvets.2022.888207)
Supplement: Supplementary file 1 [file Data_Sheet_1.ZIP › Supplementary file/miRDeep2 file.zip!/miRDeep2 file/Novel_Structure/SPLP42A1/Novel_484.pdf]

Provisional ID	:	NC_019472.2_21309
Score total	:	2
Score for star read(s)	:	-1.3
Score for read counts	:	0
Score for mfe	:	1.7
Score for randfold	:	1.6
Score for cons. seed	:	
Total read count	:	165
Mature read count	:	165
Loop read count	:	0
Star read count	:	0

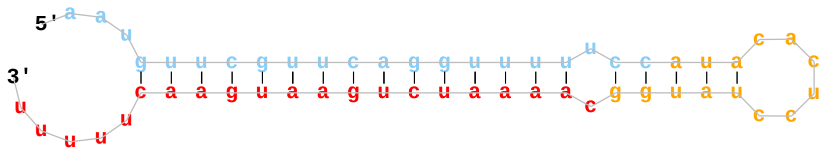

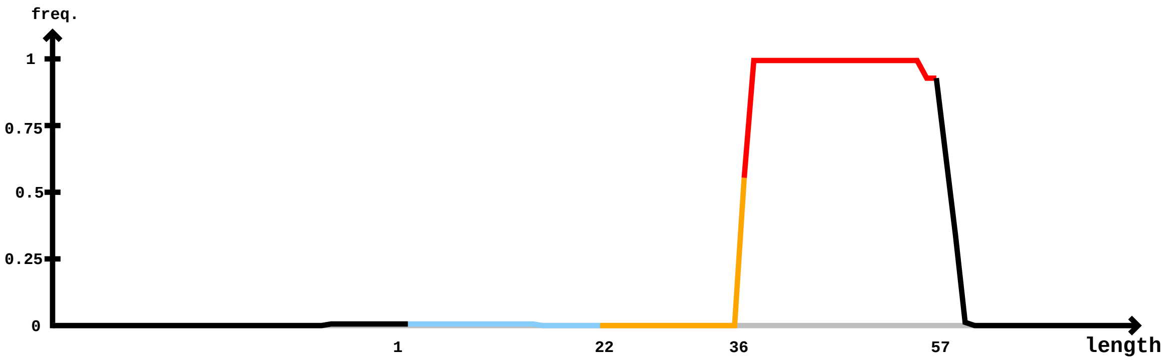


```
                                           Star                                     Mature
5'- ugaagcacaaaaagcuuauguauuggguuggccaaaauguucguucagguuuuuccauacacuccuauggcaaaaucugaaugaacuuuuugcccauuugaacucaaucac   -3'    exp
    ..((((.......)))).((.((((((((((.(((((.(((((((((((((((.(((((......))))).))))))))))))))).))))).))).....)))))))))).    reads     mm         sample
    ...........................uuggccaaaauguucguucagg..............................................................    1         0          seq
    ......................................................................Aaaaaucugaaugaacuuu......................    9         1          seq
    ......................................................................caaaaCcugaaugaacuuuuu....................    1         1          seq
    ......................................................................Aaaaaucugaaugaacuuuuu....................    80        1          seq
    ......................................................................caaaaucugaaugaacuuuuu....................    2         0          seq
    .......................................................................aaaaucugaaugaacuuu......................    2         0          seq
    .......................................................................aaaaucugaaugaaUuuuuu....................    1         1          seq
    .......................................................................aaaCucugaaugaacuuuuu....................    8         1          seq
    .......................................................................aaaaucugaaugaacCuuuug...................    1         1          seq
    .......................................................................aaaCucugaaugaacuuuuug...................    4         1          seq
    .......................................................................aaaaucugaaugaacuuuuuC...................    1         1          seq
    .......................................................................aaaaucugaaugaacuuuuugA..................    53        1          seq
    .......................................................................aaaaucugaaugaacuuuuGgc..................    1         1          seq
    .......................................................................aaaaucugaaugaacuuuuugGc.................    2         1          seq
```
